# Antitumor Activity of Anti‐miR‐21 Delivered through Lipid Nanoparticles

**DOI:** 10.1002/adhm.202202412

**Published:** 2022-12-09

**Authors:** Zhongkun Zhang, Yirui Huang, Jing Li, Fei Su, Jimmy Chun‐Tien Kuo, Yingwen Hu, Xiaobin Zhao, Robert J. Lee

**Affiliations:** ^1^ Division of Pharmaceutics and Pharmacology College of Pharmacy The Ohio State University 500 W 12th Avenue Columbus OH 43210 USA; ^2^ Zhejiang Haichang Biotechnology Co., Ltd. Hangzhou Zhejiang 310000 P. R. China; ^3^ The Whiteoak Group, Inc. Rockville MD 20855 USA

**Keywords:** cancer therapy, immunoregulation, lipid nanoparticles, oligonucleotides

## Abstract

The ability of lipid nanoparticles (LNPs) to deliver nucleic acids have shown a great therapeutic potential to treat a variety of diseases. Here, an optimized formulation of QTsome lipid nanoparticles (QTPlus) is utilized to deliver an anti‐miR‐21 (AM21) against cancer. The miR‐21 downstream gene regulation and antitumor activity is evaluated using mouse and human cancer cells and macrophages. The antitumor activity of QTPlus encapsulating AM21 (QTPlus‐AM21) is further evaluated in combination with erlotinib and atezolizumab (ATZ). QTPlus‐AM21 demonstrates a superior miR‐21‐dependent gene regulation and eventually inhibits A549 non‐small cell lung cancer growth in vitro. QTPlus‐AM21 further induces chemo‐sensitization of A549 cells to erlotinib with a combination index of 0.6 in inhibiting A549 cell growth. When systemically administers to MC38 tumor‐bearing mouse model, QTPlus‐AM21 exhibits an antitumor immune response with over 80% tumor growth inhibition (TGI%) and over twofold and fourfold PD‐1 and PD‐L1 upregulation in tumors and spleens. The combination therapy of QTPlus‐AM21 and ATZ further shows a higher antitumor response (TGI% over 90%) and successfully increases M1 macrophages and CD8 T cells into TME. This study provides new insights into the antitumor mechanism of AM21 and shows great promise of QTPlus‐AM21 in combination with chemotherapies and immunotherapies.

## Graphical abstract

QTPlus‐encapsulated AM21 shows direct tumor growth inhibition and chemo‐sensitization to erlotinib in vitro. Meanwhile, QTPlus‐AM21 also shows antitumor immunoregulation in the tumor microenvironment in vivo which benefits the anti‐PD‐L1 therapy.

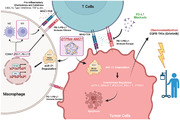

## 1 Introduction

MicroRNAs (miRNAs) are short non‐coding RNAs that can regulate gene expression through mRNA degradation or partial translational repression.^[^ ^1^ ^]^ Research showed that miRNA can not only be found inside the cell but also migrate to extracellular compartments and systemic fluids, making miRNAs efficient biomarkers in multiple diseases.^[^ ^2^ ^]^ The first miRNA used as a biomarker is miRNA‐21 (miR‐21), where researchers found that high expression of miR‐15, miR‐21, and miR‐210 in the serum of patients with diffuse large B‐cell lymphoma (DLBCL) in 2008.^[^ ^3^ ^]^ Specifically, miR‐21 exhibited the largest differential expression in clinical samples and is highly related to disease progression and patient survival. Later, miR‐21 overexpression has also been demonstrated as a promising biomarker in poor diagnosis and prognosis of many solid tumor types including non‐small cell lung cancer (NSCLC) and colorectal cancer.^[^ ^4^, ^5^ ^]^ Recently, researchers found that miR‐21 may function not only as a biomarker but also as an oncogenic miRNA that regulates the epigenetic level of cell apoptosis, DNA repair, cell proliferation, tumor metastasis, and drug resistance by downregulating tumor suppressor genes such as AKT1, DDAH1, PTEN, PDCD4, etc.^[^ ^6^ ^]^ In addition, research also showed that miR‐21 is also associated with pro‐tumor immune responses with a higher population of M2‐macrophages, suggesting that miR‐21 may induce drug resistance against not only chemotherapies but also immunotherapies.^[^ ^7^, ^8^ ^]^ Regardless of the continuous showcase of miRNA as biomarkers in cancer, however, efficient therapeutics targeting pro‐tumor miRNAs are underdeveloped.

Antisense oligonucleotide (ASO) is a single‐stranded deoxyribonucleotide that is complementary to its target. The mechanisms of antisense targeting include gene downregulations by recruiting RNAse H endonuclease activity that could cleave the heteroduplex formed by ASO and target genes^[^ ^9^ ^]^ or by sterically blocking the target RNA sequences for splicing and translational modulations.^[^ ^10^ ^]^ ASO could not only target mRNA but also oligonucleotides such as miRNA or small‐interfering RNA (siRNA), which makes it a promising approach for therapeutically inhibiting miRNA.^[^ ^9^ ^]^ However, the unmodified anti‐miRNA antisense oligonucleotides (anti‐miRs) are sensitive to nucleases that are easily cleared from systemic fluids.^[^ ^11^ ^]^ To overcome this barrier, many types of chemical modifications of anti‐miRs have been introduced.^[^ ^12^ ^]^ Modification of the phosphate backbone, the nucleic acid base, and the ribose sugar moiety has been extensively employed to improve drug pharmacokinetics, pharmacodynamics, and biodistribution.^[^ ^12^ ^]^ Chemical conjugation of anti‐miRs, including small molecules, peptides, aptamers, and antibodies, has also been developed to improve tissue‐specific biodistribution and therapeutic efficacy.^[^ ^12^ ^]^ Nonetheless, free drug of anti‐miRs contains a high density of negative charges which make them less accessible to the tumor microenvironment (TME) through cellular uptake. Therefore, an efficient delivery platform is needed for therapeutic anti‐miRs.

QTsome is a novel lipid nanoparticles (LNPs) platform using a combination of a quaternary amine‐based cationic lipid and a tertiary amine‐based ionizable lipid to facilitate drug delivery.^[^ ^13^ ^]^ The design of using a cocktail of cationic lipid and ionizable lipid in QTsome could achieve an optimal pH‐dependent drug loading and releasement profile.^[^ ^14^ ^]^ Preliminary studies have shown that the traditional design of QTsome was able to deliver oligonucleotides.^[^ ^13^ ^]^ However, traditional QTsome (QTsome Original) contains a high amount of PEG‐lipids and outdated functional lipids which impedes cellular uptake and releasement of nucleic acid cargos into the cytoplasm, making the gene delivery by QTsome Original less efficient than other emerging LNPs platforms from Moderna, Pfizer/BioNTech, Alnylam Pharmaceuticals, etc.^[^ ^15^ ^]^ Here, an optimized formulation of QTsome was utilized to deliver an anti‐miR with locked nucleic acid (LNA) modification against miR‐21 (AM21) for cancer treatment. Briefly, the rationale for optimizing traditional QTsome (QTsome Original) into QTsome Plus (QTPlus) was based on the LNPs compositions from Moderna, Pfizer/BioNTech, and Alnylam Pharmaceuticals which utilized minimal amount of PEG lipids and optimal molar ratio between PEG lipids, cationic lipids, and ionizable lipids.^[^ ^15^ ^]^ In the present study, QTPlus‐AM21 showed significant miR‐21 inhibition through downstream gene regulation compared with QTsome Original‐AM21. In addition, QTPlus‐encapsulating AM21 (QTPlus‐AM21) showed significant antitumor activity in A549 NSCLC in vitro and MC38 colorectal tumors in vivo. In the A549 NSCLC model in vitro, QTPlus‐AM21 significantly induced chemo‐sensitization with erlotinib, a tyrosine kinase inhibitor, by promoting PTEN and inhibiting EGFR expression pharmacologically. In the MC38 tumor model, QTPlus‐AM21 could induce macrophage polarization into the type 1 macrophage (M1) population with upregulated PD‐1/PD‐L1 expression in tumor and immune cells. QTPlus‐AM21 further showed enhanced antitumor immunity when systemically administered in combination with atezolizumab (ATZ), an anti‐PD‐L1 therapy, with a significant increase of antitumor immune cell populations in TME, suggesting that QTPlus‐AM21 would not only be a strong antitumor candidate directly targeting tumor cells but also be a promising immunomodulatory agent to potentiate antitumor immunity.

## 2 Experimental Section

### 2.1 Materials

1,2‐Dioleoyl‐3‐trimethylammonium‐propane (DOTAP) was purchased from MedChemExpress (Monmouth Junction, NJ). 1‐(2,3‐bis(((9Z,12Z)‐octadeca‐9,12‐dien‐1‐yl)oxy)propyl)pyrrolidine (A‐066) was purchased from Hangzhou Dragonpharm Co., Ltd (Hangzhou, China). Cholesterol was purchased from Avanti Polar Lipids, Inc. (Birmingham, AL). 1,2‐dioleoyl‐sn‐glycero‐3‐phosphoethanolamine (DOPE) and 1,2‐Dimyristoyl‐rac‐glycero‐3‐methoxypolyethylene glycol‐2000 (DMG‐PEG2000) were purchased from NOF America (Cambridge, MA). AM21 were purchased from Integrated DNA Technologies, Inc. (Coralville, IA) (16‐mer AM21 sequence: 5’‐A*+T*+C*A* G*+T*+C*+T*G*A*+T*A*A*G*+C*+T‐3’. Scramble 16‐mer oligonucleotide sequence: 5’‐+C*A*C*G*+T*+C*+T*A*+T*A*+C*G*+C*+C*+C*A*‐3’. “+” represents locked nucleic acid, LNA, bases. “*” represents phosphorothioated backbone). Erlotinib was purchased from Cayman Chemical (Ann Arbor, MI). ATZ was kindly provided by the Arthur G. James Cancer Hospital from The Ohio State University (Columbus, OH).

### 2.2 QPlus‐AM21 Formulation and Characterization

Empty QTsome Original and QTPlus were prepared by hand‐rapid injection of the lipid mixture into the acetic acid buffer. Detailed compositions of QTsome Original and QTPlus can be found in **Table** **1**. As an example of QTPlus, DOTAP, A‐066, DOPE, cholesterol, and DMG‐PEG2000 were prepared at a molar ratio of 1.5/50/12/35/1.5 in ethanol. AM21 and scramble oligonucleotide solutions were prepared in DEPC‐treated water. Oligonucleotide solutions were mixed with empty QTsome Original or QTPlus phase with equal volume to reach the final lipid‐to‐oligo ratio at 10/1 (w/w). The final lipid concentration of QTsome was 10 mg mL^−1^, and the final oligonucleotide concentration was 1 mg mL^−1^. The final product of QTsome encapsulating oligonucleotides was further titrated from pH 5.5 to pH 7.4. Particle sizes and zeta potentials of QTsome‐encapsulating oligonucleotides were measured by dynamic light scattering (DLS) using a NICOMP NANO ZLS Z3000 (Entegris, Billerica, MA). Gel electrophoresis was performed using 1% agarose gel loaded with 1 ug oligonucleotide per well. Fluorescence imaging of ethidium bromide was taken after 20 min of gel electrophoresis at 100 V. Cryo‐EM images of QTPlus‐AM21 were obtained from the Center for Electron Microscopy and Analysis at the Ohio State University (Columbus, OH).

**Table 1:** Detailed compositions of QTsome Original and QTPlus

QTsome original		QTPlus	
Components	Molar ratio [%]	Components	Molar ratio [%]
DOTAP	5	DOTAP	1.5
DODMA	40	A‐066	50
DOPC	27.5	DOPE	12
Cholesterol	20	Cholesterol	35
DMG‐PEG 2000	7.5	DMG‐PEG 2000	1.5

### 2.3 Cell Culture

RAW 264.7 murine macrophage cell line and MC38 murine colorectal carcinoma cell lines were kind gifts given by Dr. Peixuan Guo and Dr. Christopher Coss at The Ohio State University College of Pharmacy, respectively. THP‐1 human monocyte cell line was kindly provided by Dr. Joshua Englert at The Ohio State University Wexner Medical Center. KB, A549, and Hep3B cell lines were purchased from Millipore Sigma (Burlington, MA). RAW 264.7 and MC38 were grown in DMEM supplemented with 10% FBS and 1*x* antibiotic‐antimycotic. THP‐1, KB, and A549 were grown in RPMI supplemented with 10% FBS. Hep3B was grown in MEM supplemented with 10% FBS. Cells were maintained at 37 °C and grown under a humidified atmosphere containing 5% CO_2_.

### 2.4 In Vitro Gene Regulation

KB, A549, Hep3B, THP‐1, MC38, and RAW264.7 cells were seeded at 3 × 10^5^ cells per well in 6‐well plates 24 h before treatments. Cells were treated with scramble oligonucleotide or AM21 in free solution, lipofectamine, QTsome Original, and QTPlus formulations. For KB, A549, and Hep3B cell lines, cells were treated with 200 nm of scramble oligonucleotide or AM21 for 4 h and then washed three times with PBS. Cells were further incubated with fresh complete medium for 16–24 h at 37 °C. For MC38, RAW264.7, and THP‐1 cell lines, cells were treated with 400 nm of scramble oligonucleotide or AM21 for overnight incubation at 37 °C. Total RNA was extracted using TRI reagent (Zymo Research) per manufacturer protocol. cDNA was prepared by high‐capacity cDNA reverse transcription kit (Invitrogen, Waltham, MA, USA), and real‐time qPCR (RT‐qPCR) was done using SsoAdvanced Universal SYBR Green Supermix (Bio‐Rad Laboratories, Hercules, CA) on a QuantStudio 7 Flex Real‐time PCR System. All the RT‐qPCR primers were purchased from Sigma‐Aldrich (Burlington, MA). Beta‐actin (Actb) was selected as the housekeeping gene control. The relative amount of RNA level was calculated and compared according to the 2‐ΔΔCt method.^[^ ^16^ ^]^

### 2.5 Colony Formation Assay

A549 cells were seeded at 100 cells per well in 24‐well plates 24 h before treatments. Cells were treated with AM21 in free solution and QTPlus formulation at concentrations of 0, 1, and 10 um. Cells were allowed to form colonies for up to 1‐week followed by fixation in methanol and colony detection by crystal violet. The cell colonies stained with crystal violet were further dissolved in DMSO and absorbance was read at 570 nm to calculate the percentage of colony formation compared to the untreated groups.

### 2.6 MTS Assay

A549 cells were seeded at 3000 cells per well in 96‐well plates 25 h before treatments. Cells were treated with QTPlus AM21 (concentrations from 0 nm to 6.4 um), erlotinib (0–200 um), and a combination of QTPlus AM21 and erlotinib at a fixed concentration ratio (QTPlus AM21/erlotinib of 6.4/200). After 72‐h treatment, cell viability was examined by CellTiter 96 AQueous One Solution (Promega, Madison, WI) per manufacturer protocol. The synergistic effects of QTPlus‐AM21 and erlotinib were determined by CompuSyn software (The ComboSyn, Inc.).

### 2.7 Macrophage‐Tumor Cell Co‐Culture Study Wound Healing Assay

MC38 and RAW264.7 cells were seeded on 6‐well plates in a total number of 6 × 10^6^ cells per well with a fixed macrophage‐to‐tumor cell ratio of 3/1. Cells were treated with 400 um of AM21 in free solution or QTPlus with or without 1 ug mL^−1^ of lipopolysaccharide (LPS) stimulation. Cells were incubated for 24 h at 37 °C and collected for flow cytometry analysis.

For the wound healing study, a scratch wound healing model was conducted to examine the migratory ability of MC38 cells in the presence of macrophages following treatment. A scratch wound across the well was made using a 10 uL pipet tip immediately before treatment. Cells were washed by PBS and incubated with complete media containing 400 um of AM21 in free solution and QTPlus with or without 1 ug mL^−1^ of LPS stimulation. Cells were allowed to proliferate at 37 °C for 24 h. Distances between the edges of the wound were measured by a Nikon Eclipse Ti‐S microscope (Nikon, Tokyo, Japan).

### 2.8 Enzyme‐Linked Immunosorbent Assay (ELISA)

Human PTEN Matched Antibody Pair Kit was purchased from Abcam (Cambridge, UK). Human EGFR Matched ELISA Antibody Pair Set was purchased from Sino Biological (Beijing, China). A549 cells were seeded at 8 × 10^5^ cells per plate in 60 mm culture dishes 24 h before treatment. Cells were treated with 10 ug of QTPlus‐AM21 and 20 um of erlotinib individually or in combination. After overnight treatment, cells were harvested and homogenized in Pierce RIPA buffer (Thermo Fisher Scientific). Total proteins were extracted after incubating on ice for 30 min and centrifuged at 14 000 × *g* for 30 min at 4 °C. Protein concentrations were quantified and unified by Pierce BCA Protein Assay Kit (Themo Fisher Scientific). PTEN and EGFR concentrations were measured per the manufacturer's protocol.

### 2.9 In Vivo Antitumor Efficacy Study

To study the synergistic mechanism of QTPlus‐AM21 and erlotinib, the A549 xenograft mouse model was generated by subcutaneously inoculating nude mice with 2.5 × 10^6^ cells per mouse on the right flank. Treatments were initiated once tumors reached 50–100 mm^3^. Mice (*n* = 3) were intravenously treated with saline, 3 mg kg^−1^ of QTPlus‐encapsulating scramble oligonucleotide (QTPlus Ctrl), 3 mg kg^−1^ of QTPlus‐AM21, orally treated with 50 mg kg^−1^ erlotinib, or QTPlus‐AM21/erlotinib combination (3 mg kg^−1^ of QTPlus‐AM21 and 50 mg kg^−1^ erlotinib). All mice were dosed every 3 days for 5 doses. Tumor tissues were harvested within 24 h after the final dose. Tumor tissues from nude mice were immediately lysed to collect protein samples for ELISA assays to determine the PTEN and EGFR concentrations in TME.

To evaluate the combination therapy of QTPlus‐AM21 and anti‐PD‐L1 therapy, the MC38 murine colorectal syngeneic model was generated by subcutaneously inoculating C57BL/6 mice (obtained from Charles River Laboratories) with 1 × 10^6^ cells per mouse on the right flack. Treatments were initiated once tumors reached 50–100 mm^3^. In the dose selection study, mice (*n* = 3) were intravenously treated with saline, 3 mg kg^−1^ of QTPlus Ctrl, 3 mg kg^−1^ of QTPlus‐AM21, and 6 mg kg^−1^ of QTPlus‐AM21. In the antitumor efficacy study to evaluate the synergistic effect between QTPlus‐AM21 and ATZ, mice (*n* = 5) were intraperitoneally treated with saline, 3 mg kg^−1^ QTPlus‐Ctrl, 3 mg kg^−1^ QTPlus‐AM21, 10 mg kg^−1^ ATZ, and QTPlus‐AM21/ATZ combination (3 mg kg^−1^ QTPlus‐AM21 and 10 mg kg^−1^ ATZ).

All mice were dosed every 3 days for 5 doses. Tumor growth and body weight were monitored, and the tumor volumes were calculated according to the formula:

(1) $$\mathrm{Tumor}\;\mathrm{Volume}=\frac{\mathrm{Length}\;\times {\mathrm{Width}}^{2}}{2}$$

All animal studies were reviewed and approved by The Ohio State University Institutional Laboratory Animal Care and Use Committee (IACUC) (Animal Welfare Assurance Number: D16‐00168 (A3261‐01)). All mice were euthanized one day after the last dose to peak the immune activation and protein expression in TME. Terminal Tumor growth inhibition (%TGI) was determined by the formula:

(2) $$\%\mathrm{TGI}\;=\;\frac{1-\left(T_{t}/T_{0}\right)/\left(C_{t}/C_{0}\right)}{1-C_{0}/C_{t}}\times \;100\%$$

where *T*_t_ stands for average tumor volume of treatment group at the day of measurement, *T*_0_ stands for average tumor volume of treatment group at day 0, *C*_t_ stands for average tumor volume of the control group at the day of measurement, and *C*_0_ stands for average tumor volume of the control group at day 0. %TGI > 50% was considered meaningful.

Tumor and spleen tissues from C57BL/6 mice were harvested within 24 h after the final dose. Tumors and spleens were homogenized in TRI reagent using probe sonication, and total RNA was extracted per the manufacturer's protocol for RT‐qPCR. Tumor tissues were further smashed by a 5‐mL syringe through a 70 µm cell strainer to generate single‐cell suspensions for flow cytometry analysis.

### 2.10 Flow Cytometry

Alexa Fluor 700 anti‐mouse CD45 (30‐F11), APC/Cyanine7 anti‐mouse CD3e (145‐2C11), FITC anti‐mouse CD4 (RM4‐5), PE/Cyanine7 anti‐mouse CD8a (53‐6.7), PE anti‐mouse FOXP3 (MF‐14), and BV‐650 anti‐mouse NK1.1 (PK136) were used for detection of T cells and NK cells populations in the mouse tumor. FITC anti‐mouse F4/80 (BM8), PE anti‐mouse CD206 (C068C2), BV‐605 anti‐mouse CD86 (PO3), APC‐Cy7 anti‐mouse CD11b (M1/70), and BV‐650 anti‐mouse Gr‐1 (RB6‐8C5) were used for detection of macrophages and myeloid‐derived suppressor cells (MDSCs) populations in the mouse tumor. All the fluorophore‐conjugated antibodies and True‐Nuclear Transcription Factor Buffer Set for FOXP3 staining were purchased from BioLegend (San Diego, CA, USA). Single‐cell suspensions of tumor tissues in FACS staining buffer were stained per manufacturer protocol. Stained cells were analyzed using a BD LSR II or Fortessa flow cytometer in Flow Cytometry Shared Resources (FCSR) at The Ohio State University Comprehensive Cancer Center.

### 2.11 Statistical Analysis

All studies were done in triplicate unless otherwise specified. Data are presented as means ± standard deviations unless otherwise indicated. Statistical analysis was conducted using Microsoft Excel. One‐way ANOVA was used to determine variances in means between two or more treatment groups. Tukey Honestly Significant Difference (HSD) test was further used as a post‐hoc analysis to determine statistically significant difference after one‐way ANOVA. Student's *t*‐test was performed in case of comparing the statistical differences between two groups of interest only. A *p*‐value of 0.05 was selected as the cutoff for statistical significance.

## 3 Results

### 3.1 QTPlus as a Platform to Deliver AM21 for miR‐21 Inhibition

**Figure 1:**
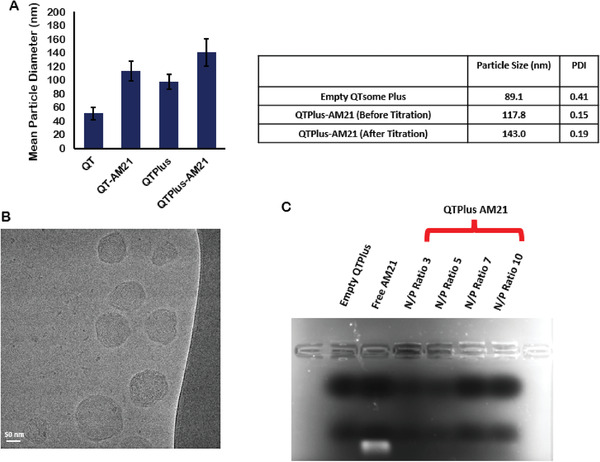
Particle characterization of QTPlus‐AM21. A) Particle sizes of empty QTsome Original, QTsome Original‐AM21, empty QTPlus, and QTPlus‐AM21 (*n* = 3); B) Cryo‐EM image of QTPlus‐AM21; C) 1% Agarose gel electrophoresis of QTPlus‐AM21 with different nitrogen‐to‐phosphate (N/P) ratio.

Particle sizes of the empty QTPlus and the QTPlus‐AM21 were larger than the empty QTsome Original and the QTsome Original‐AM21 which are 80–90 nm (empty QTPlus) and 110–120 nm (QTPlus‐AM21) (**Figure** **1**A). In addition, the particle sizes of QTPlus‐AM21 also slightly increased to 140 nm after titrating the final product from pH 5.5 to a pH 7.4 (Figure 1A). The surface charges of QTPlus‐AM21 measured as zeta potentials were 22.07 mV ± 2.56 before titration and 2.20 mV ± 0.34 after titration. QTPlus‐AM21 exhibited an onion‐like structure with multilamellar layers composed of lipids and ASOs (Figure 1B) and was capable to encapsulate any amount of oligonucleotide of N/P ratio from 3 to 10 (Figure 1C).

**Figure 2:**
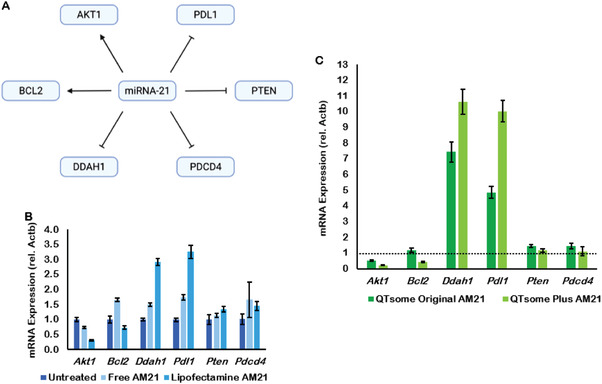
miR‐21 and AM21 gene regulation in human cancer cells. A) miR‐21 downstream gene signaling. KB and A549 cells were treated with AM21 at 200 nm for 4 h followed by replacement with fresh complete media and incubation for overnight. AM21 regulated miR‐21 downstream genes in free solution and lipofectamine in B) KB cells, in QTsome Original and QTPlus‐AM21 in C) A549 cells (*n* = 3).

Many literatures suggest that miR‐21 expression is highly associated with its downstream genes including Akt1, Bcl2, Ddah1, Pdcd4, Pd‐l1, and Pten, and inhibiting miR‐21 would greatly induce cancer cell apoptosis by regulating its downstream genes.^[^ ^13^, ^17^, ^18^, ^19^, ^20^ ^]^ In KB and A549 cells, AM21 consistently induced Akt1 downregulation, Ddah1 upregulation, and PD‐L1 upregulation in free solution, lipofectamine, and QTsome formulation (**Figure** **2**B,C). However, the roles of AM21 in regulating Bcl2, Pten, and Pdcd4 expression are controversial when AM21 is transfected by free solution and QTsome Original or QTPlus to cells (Figure 2B,C). Based on these profiles, three human cancer cell lines (KB, A549, Hep3B) were treated with QTPlus‐AM21 to select the most sensitive cancer cell models for miR‐21 inhibition. QTPlus‐AM21 showed the highest gene regulation levels in A549 cells compared with KB and Hep3b cells in vitro (**Table** **2**). In addition, QTPlus‐AM21 exhibited much more significantly miR‐21 downstream gene regulations compared with QTsome Original‐AM21 in the A549 cell line in vitro (Figure 2C).

**Table 2:** miR‐21 downstream gene regulation by QTPlus‐AM21 in KB, A549, and Hep3B cell lines (*n* = 3)

miR21 downstream genes of interest	KB	A549	Hep3b
	mRNA expression ± STD		
Akt1	0.89 ± 0.10	0.22 ± 0.02	1.67 ± 0.30
Bcl2	1.20 ± 0.11	0.44 ± 0.05	0.89 ± 0.23
Ddah1	1.27 ± 0.12	10.64 ± 0.80	3.68 ± 0.94
Pd‐l1	0.99 ± 0.12	10.03 ± 0.69	4.07 ± 1.94
Pten	1.08 ± 0.10	1.16 ± 0.11	0.71 ± 0.14
Pdcd4	1.25 ± 0.15	1.10 ± 0.28	0.68 ± 0.18

### 3.2 Synergistic Antitumor Effect between QTPlus‐AM21 and Erlotinib

**Figure 3:**
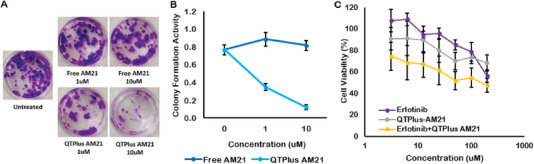
QTPlus‐AM21 inhibits A549 cell growth in vitro. A) Colony formation assay of A549 cells treated with AM21 (0, 1, and 10 um) in free solution and QTPlus formulation for 1 week. B) The percentage of colony formation was determined by UV–vis spectrometry at 540 nm (crystal violet). C) Cell viability assay of A549 cells treated with erlotinib single treatment (0, 3.13, 6.25, 12.5, 25, 50, 100, 200 µm), QTPlus‐AM21 single treatment (0, 100, 200, 400, 800, 1600, 3200, 6400 nm) or in combination with QTPlus‐AM21 with a fixed concentration ratio (QTPlus AM21/erlotinib of 6.4/200) for 72 h (*n* = 3).

**Figure 4:**
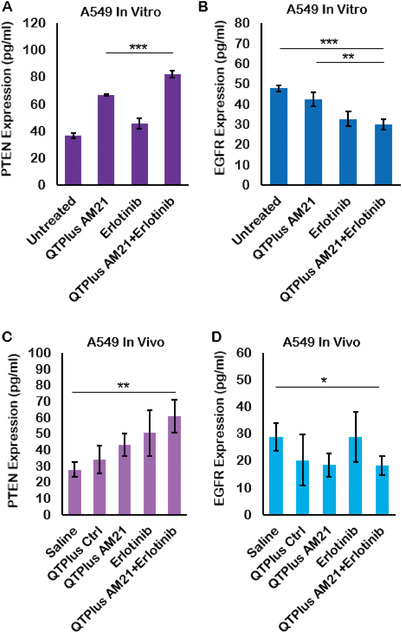
PTEN and EGFR concentration in A549 tumor model treated with QTPlus‐AM21 and erlotinib A,B) in vitro and C,D) in vivo (*n* = 3). A549 cells were treated with 10 ug of QTPlus‐AM21 and 20 um of erlotinib individually or in combination overnight. A549 tumors were collected from tumor‐bearing nude mice after treated with 2 mg kg^−1^ QTPlus‐AM21 and 50 mg kg^−1^ of erlotinib individually or in combination every 3 days for 5 doses. Protein samples from treated A549 cells and collected tumors were collected for ELISA assay. Student's *t*‐test was performed to determine the statistical differences between QTPlus‐AM21+Erlotinib group versus the untreated or saline group and between QTPlus‐AM21+Erlotinib group versus QTPlus‐AM21 group. *p < 0.05, **p < 0.01, ***p < 0.001.

When AM21 was long‐termly treated in A549 cells to evaluate the colony formation of A549 human cancer cells, QTPlus‐AM21 exhibited a much higher inhibitory effect in colony formation compared with free AM21 (**Figure** **3**A,B). Interestingly, QTPlus‐AM21 could also sensitize A549 cells to the erlotinib‐induced cytotoxicity when QTPlus‐AM21 was treated together with erlotinib at a fixed concentration ratio (QTPlus AM21/erlotinib of 6.4/200) (Figure 3C). The combination index between QTPlus‐AM21 and erlotinib was 0.60, and the dose reduction index (DRI) for QTPlus‐AM21 and erlotinib were 6.26 and 2.26 respectively. Combination treatment of QTPlus‐AM21 and erlotinib also enhanced PTEN expression (**Figure** **4**A) and inhibited EGFR expression (Figure 4B) pharmacologically in vitro based on ELISA results. Similar PTEN upregulation and EGFR inhibition was also observed in tumor tissues from A549 tumor‐bearing nude mice treated with QTPlus‐AM21 and erlotinib in combination (Figure 4C,D) with QTPlus Ctrl not exhibiting any regulations on PTEN and EGFR expressions (Figure 4C,D).

### 3.3 Immunoregulation by QTPlus‐AM21

**Figure 5:**
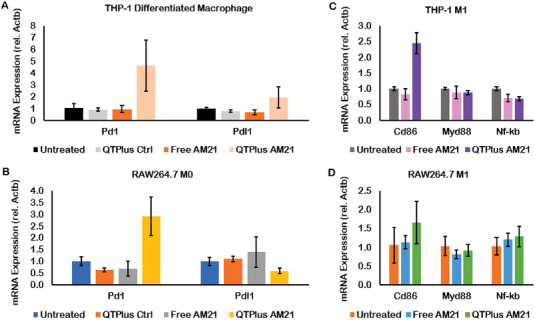
QTPlus‐AM21 regulated PD‐1/PD‐L1 expression in A) THP‐1 cells and B) RAW264.7 cells and induced M1 polarization in C) THP‐1 and D) RAW264.7 cell lines (*n* = 3). Human macrophages differentiated from THP‐1 cells and mouse RAW264.7 macrophages were treated with Free AM21 or QTPlus‐AM21 at 400 nm overnight in the presence or absence of 1 ug mL^−1^ of LPS for further M1 polarization.

**Figure 6:**
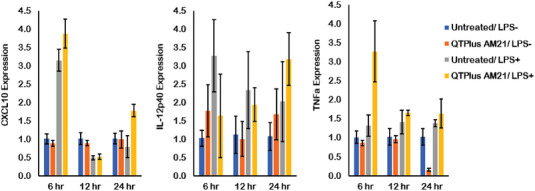
QTPlus‐AM21 induced pro‐inflammatory cytokines and chemokines in LPS‐stimulated RAW264.7 cells in vitro (*n* = 3). RAW264.7 cells were treated with 400 nm of QTPlus‐AM21 overnight in the presence or absence of 1 ug mL^−1^ of LPS for M1 polarization. Expression of CXCL10, IL‐12p40, and TNFa was measured by RT‐qPCR.

**Figure 7:**
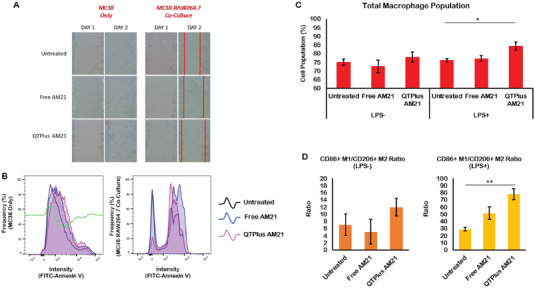
QTPlus‐AM21 regulated macrophage to inhibit tumor growth in vitro. Mouse MC38 cells and RAW264.7 cells were co‐cultured with a fixed macrophage‐to‐tumor cell ratio of 3/1 followed by treatment with 400 nm of QTPlus‐AM21 overnight in the presence of or absence of LPS for M1 polarization. A) RAW264.7 cells treated with QTPlus‐AM21 inhibited wound‐healing by MC38 colorectal cancer cells; B) QTPlus‐AM21 regulated RAW264.7 cells to enhance apoptosis in MC38 cells. QTPlus‐AM21 induced C) macrophage proliferation and D) polarization to M1 population. The green line in (B) indicates the plot differences in fluorescent intensity of FITC‐conjugated Annexin V from the untreated group to QTPlus‐AM21 group. Student's *t*‐test was performed to determine the statistical differences between QTPlus‐AM21 group versus the untreated in the presence of LPS (*n* = 3). *p < 0.05, **p < 0.01.

In immune system, miR‐21 inhibition may polarize macrophages into tumoricidal M1 population.^[^ ^8^ ^]^ MiR‐21 inhibition is also associated with the expressions of certain pro‐inflammatory cytokines and chemokines, including IL‐12, CXCL10, TNFa, which would facilitate M1 macrophages to improve the antitumor T‐cell responses.^[^ ^21^ ^]^ However, miR‐21 expression is also inversely associated with PD‐1/PD‐L1 expression where inhibiting miR‐21 may promote PD‐1/PD‐L1 expression to facilitate immune escape between immune cells and tumor cells.^[^ ^17^ ^]^ Here in the present study, PD‐1 upregulation was observed by RT‐qPCR in naïve human (differentiated from THP‐1) and murine (RAW264.7) macrophages without LPS stimulation after treated with 400 nm of QTPlus‐AM21 for 24 h (**Figure** **5**A,B). In addition, QTPlus‐AM21 could enhance Cd86 upregulation in both human and murine macrophage cell lines when they are polarized into M1 population by LPS stimulation (Figure 5C,D). Exogenous oligonucleotides or lipids may also act as antigens to randomly stimulate macrophages by binding with toll‐like receptors and proliferating Myd88 and NF‐*κ*b signaling.^[^ ^22^, ^23^ ^]^ Here, free AM21 and QTPlus Ctrl did not regulate expressions of Myd88 and NF‐*κ*b (Figure 5). The activated M1 RAW264.7 cells treated with QTPlus‐AM21 also enhanced Cxcl10, Il‐12p40, and TNFa expression in vitro (**Figure** **6**). When mouse macrophages RAW264.7 were co‐cultured with mouse colorectal cancer cells MC38, treatment with QTPlus‐AM21 could increase macrophage proliferation and polarization in M1 population (**Figure** **7**C,D). Such macrophage polarization by QTPlus‐AM21 is also associated with increased apoptosis in the MC38 cancer cell population (Figure 7B) which eventually decreased MC38 growth and its wound‐healing effects in vitro (Figure 7A).

**Figure 8:**
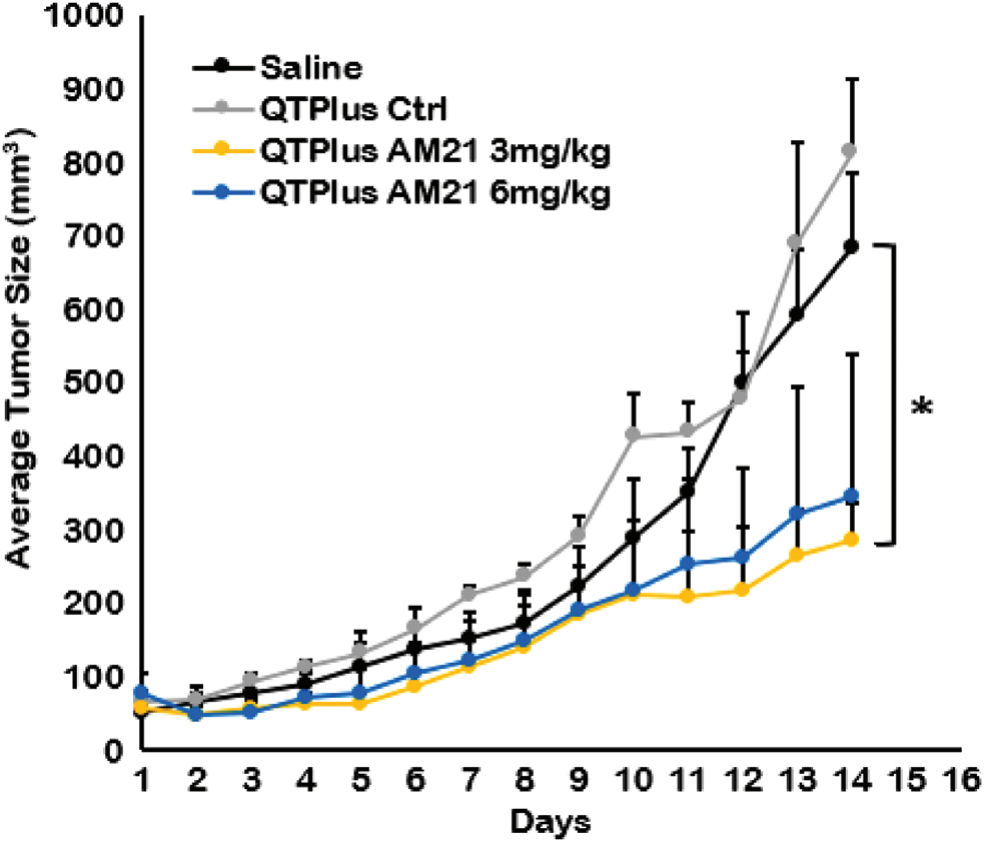
In vivo dose‐selection study of QTPlus‐AM21 based on the antitumor activities in MC38 syngeneic mouse model (*n* = 3). MC38 tumor‐bearing mice were treated with saline, QTPlus Ctrl, 3 and 6 mg kg^−1^ of QTPlus‐AM21 every 3 days for 5 doses. One‐way ANOVA was performed to determine variances in means of terminal tumor sizes among the saline group and all treated groups. Tukey HSD test was further used as a post‐hoc analysis to determine the statistical differences in the mean of terminal tumor sizes between QTPlus AM21 3 mg kg^−1^ group versus saline group. *p < 0.05.

**Figure 9:**
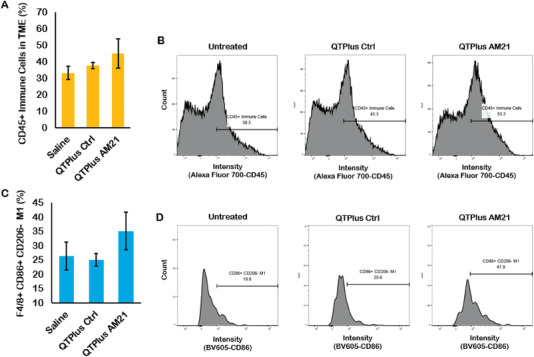
Immune cell populations in the TME from MC38 tumor‐bearing mice treated with QTPlus Ctrl and 3 mg kg^−1^ QTPlus‐AM21. A,B) QTPlus‐AM21 increased CD45+ tumor‐infiltrated immune cells and C,D) CD86+ M1 population in tumor microenvironment from MC38 syngeneic mouse model (*n* = 3). The green line in (B) indicates the plot differences in fluorescent intensity of Alexa Fluor 700‐conjugated CD45 from saline group to QTPlus‐AM21 group.

**Figure 10:**
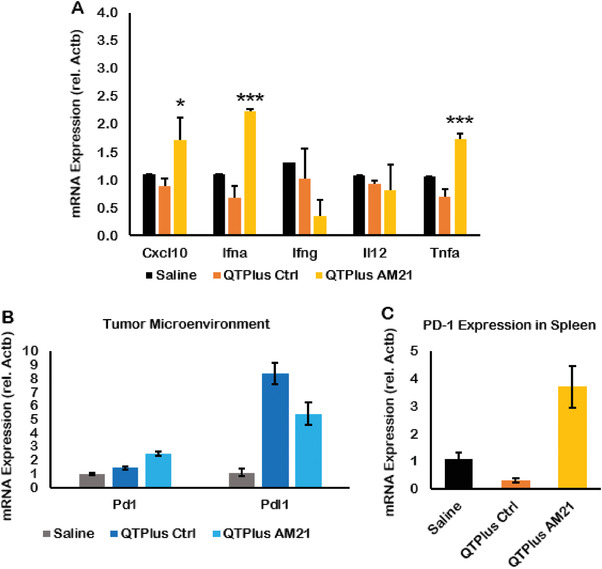
Gene expression of cytokines and immune checkpoints in MC38 tumor‐bearing mice treated with QTPlus Ctrl and 3 mg kg^−1^ QTPlus‐AM21. In MC38 syngeneic mouse model, QTPlus‐AM21 induced A) CXCL10, IFNa, and TNFa in spleen tissues. QTPlus and QTPlus‐AM21 regulated PD‐1/PD‐L1 expression in B) tumor and C) spleen tissues (*n* = 3). Student's *t*‐test was performed to determine the statistical differences in CXCL10, IFNa, and TNFa expressions between QTPlus‐AM21 3 mg kg^−1^ group versus saline group. *p < 0.05, ***p < 0.001.

In the dose selection study of QTPlus‐AM21 in the syngeneic mouse model, QTPlus‐AM21 also exhibited antitumor activity in MC38 colorectal tumors (Figure 8) Mice treated with 3 mg kg^−1^ of QTPlus‐AM21 exhibited final TGI% of 68.33% ± 12.4. However, the antitumor activity by QTPlus‐AM21 was not significantly improved when the dose was increased from 3 to 6 mg kg^−1^ (**Figure** **8**). In the TME of MC38 tumor‐bearing mice, treatment with QTPlus‐AM21 showed increased CD45+ tumor‐infiltrated immune cells (**Figure** **9**A,B) and F4/80+ CD86+ M1 populations (Figure 9C,D). Significant upregulation of CXCL10, IFNa, and TNFa was also observed in spleens from mice treated with QTPlus‐AM21 (**Figure** **10**A). However, IL‐12 expression was not affected by QTPlus‐AM21 in vivo compared with in vitro results (Figures 10A and 6). PD‐1/PD‐L1 upregulation was also observed in tumor (Figure 10B) and spleen (Figure 10C) tissues from mice treated with QTPlus‐AM21 which corresponds with the results that QTPlus‐AM21 could induce PD‐1/PD‐L1 upregulation in human cancer cell lines (Figure 2) and macrophage cell lines (Figure 5A,B).

### 3.4 Combination Therapy of QTPlus‐AM21 and ATZ

**Figure 11:**
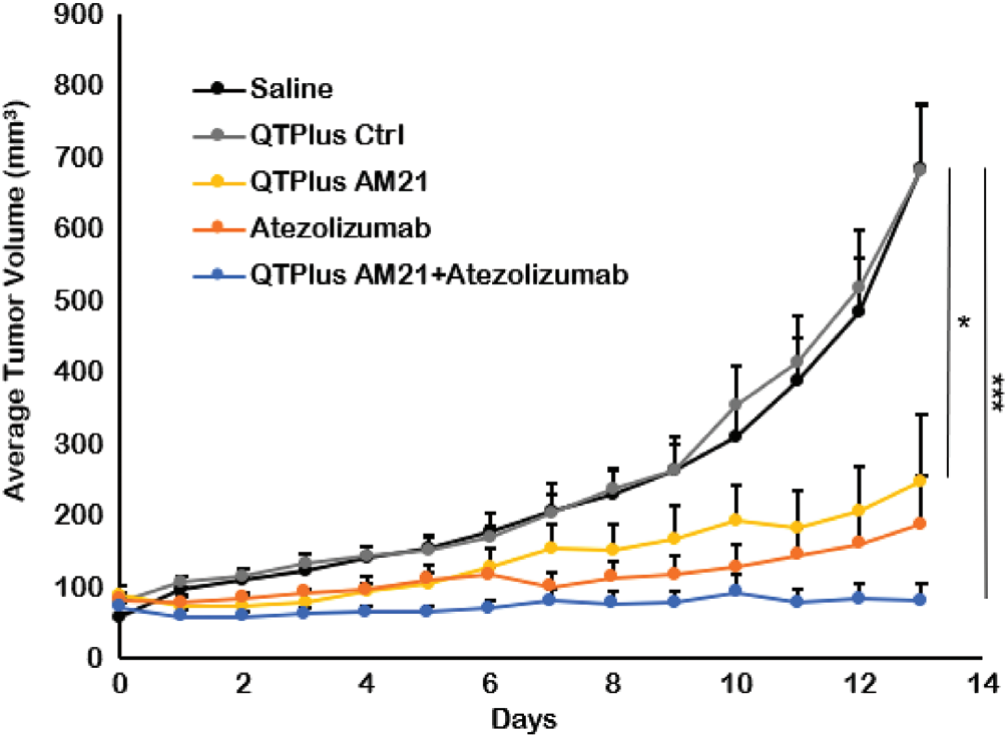
In vivo antitumor activity of QTPlus‐AM21 and ATZ in MC38 syngeneic mouse model (*n* = 5). MC38 tumor‐bearing mice were treated with saline, 3 mg kg^−1^ QTPlus‐Ctrl, 3 mg kg^−1^ QTPlus‐AM21, 10 mg kg^−1^ ATZ, and QTPlus‐AM21/ATZ combination (3 mg kg^−1^ QTPlus‐AM21 and 10 mg kg^−1^ ATZ) every 3 days for 5 doses. One‐way ANOVA was performed to determine variances in means of terminal tumor sizes among saline group and all treated groups. Tukey HSD test was further used as a post‐hoc analysis to determine the statistical differences in the mean of terminal tumor sizes between QTPlus‐AM21+ATZ group or QTPlus‐AM21 group versus the saline group. *p < 0.05, ***p < 0.001.

**Figure 12:**
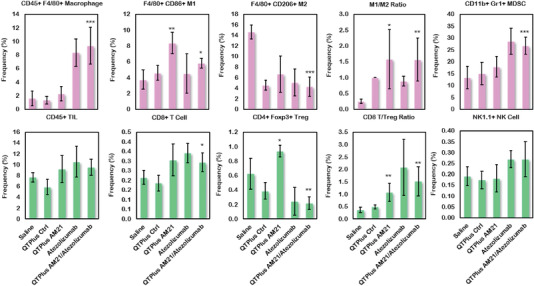
Regulation of immune cell populations in tumor microenvironment from MC38 syngeneic mouse model after treatment of QTPlus AM21 and ATZ individually or in combination (*n* = 5). Student's *t*‐test was performed to determine the statistical differences in immunes cell populations between QTPlus‐AM21 or QTPlus‐AM21+ATZ versus saline group. *p < 0.05, **p < 0.01, ***p < 0.001.

In the MC38 syngeneic mouse model, both QTPlus‐AM21 monotherapy and QTPlus‐AM21/ATZ combination showed significant antitumor responses (**Figure** **11**). The combination therapy of QTPlus‐AM21 with ATZ showed the highest TGI% of 98.8% ± 6.8 compared with QTPlus‐AM21 monotherapy (TGI% 83.5% ± 22.0) and ATZ monotherapy (88.0% ± 16.9). No statistical significance was found between QTPlus‐AM21+ATZ combination therapy and ATZ monotherapy. Nonetheless, none of the mice treated with QTPlus‐AM21+ATZ combination developed large tumors compared to mice treated with either QTPlus‐AM21 or ATZ alone (Figure S3, Supporting Information), which eventually leads to a smaller mean in terminal tumor sizes in mice treated with QTPlus‐AM21+ATZ combination. Treatment with QTPlus Ctrl did not exhibit any antitumor responses (Figures 8 and 11) nor immunogenicity by cytokine expressions (Figure 10A). However, QTPlus Ctrl was able to regulate PD‐1/PD‐L1 expressions in TME and spleens as well as decrease F4/80+ CD206+ M2 populations in TME (**Figure** **12**). In terms of the immunoregulatory crosstalk between QTPlus‐AM21 and ATZ in TME, QTPlus‐AM21 and ATZ exhibited different profiles in regulating tumor‐infiltrated immune cell populations (Figure 12). QTPlus‐AM21 was demonstrated to increase the F4/80+ CD86+ M1, CD8+ T cells, and CD4+ Foxp3+ regulatory T cells (Tregs) populations (Figure 12) and decrease the F4/80+ CD206+ M2 populations (Figure 12). ATZ was able to increase the CD45+ F4/80+ total macrophages, CD11b+ Gr‐1+ MDSCs, CD8+ T cell, NK1.1+ NK cell populations (Figure 12) and decrease the F4/80+ CD206+ M2 and CD4+ Foxp3+ Tregs populations (Figure 12). Taking together, the combination therapy of QTPlus‐AM21 and ATZ was able to significantly increase M1/M2 and CD8 T/Tregs ratios and increase CD11b+ Gr‐1+ MDSCs and NK1.1+ NK cells population in TME from MC38 tumor‐bearing mice (Figure 12).

## 4 Discussion

### 4.1 QTPlus as an Efficient Platform to Deliver AM21

LNPs have been developed as a promising platform to deliver a variety of therapeutic agents since the 1960s.^[^ ^24^, ^25^ ^]^ Liposomes were first developed to encapsulate small‐molecule chemotherapies as anticancer therapeutics.^[^ ^26^ ^]^ The benefits of using LNPs to deliver small molecules are to enhance the therapeutic efficacy by increasing the half‐life of active compounds in systemic fluids and targeting tumors through the enhanced permeability and retention (EPR) effect.^[^ ^27^, ^28^ ^]^ To maintain these advantages, the components of traditional LNPs for small molecule delivery usually contain certain amounts of PEG lipids to prevent the degradation LNPs/drug complex in systemic fluids and to reduce particle size for the EPR effect.^[^ ^27^ ^]^ However, these designs for traditional LNPs may not be capable of delivering nucleic acid payloads since nucleic acid delivery requires a rapid drug release from LNPs to the cytoplasm to achieve therapeutic gene regulation or expression.^[^ ^29^ ^]^ Therefore, when applied for nucleic acid delivery, LNPs are expected to prevent degradation in systemic fluids but to rapidly release their cargos by endosomal escape once they are internalized by cells,^[^ ^30^ ^]^ suggesting that lower amounts of PEG lipids and better functional lipids should be considered when designing LNPs for nucleic acid delivery. Moreover, particle sizes of LNPs could influence mechanisms for the cellular uptake of LNPs.^[^ ^31^ ^]^ In general, particles smaller than 200 nm are able to enter cells through clathrin‐ or caveolin‐mediated endocytosis.^[^ ^32^ ^]^ Particles larger than 500 nm may enter the cells through phagocytosis, and large particles could also be recognized by macrophages to elicit unnecessary immune responses in human bodies.^[^ ^33^ ^]^ In addition, a more positive surface charge on LNPs resulted in a more efficient cellular uptake.^[^ ^34^ ^]^ However, LNPs with extremely high surface charges also correlate with greater cytotoxicity in nonphagocytic cells which is not preferred when considering the safety of LNPs as drug delivery platforms.^[^ ^35^ ^]^ In vivo, positively charged particles have been shown to selectively target the neovasculature of the tumor.^[^ ^36^ ^]^ Here, the final product of QTPlus‐AM21 with a mean particle size of around 140 nm and a slightly positive surface charge of 2.2 mV would be considered suitable for cellular uptake and AM21 delivery. When QTPlus‐AM21 was formulated at lower final concentrations of AM21 (0.25 mg mL^−1^) and lipids (2.5 mg mL^−1^) while maintaining the same lipid‐to‐oligo ratio of 10/1 (w/w), the final particle size was reduced to 117.5 nm ± 0.75 with a PDI of 0.019. This suggests that the formulation of QTPlus‐AM21 prepared at reduced concentrations could yield smaller and more consistent particle sizes with smaller PDI, which are preferred for large‐scale manufacturing and clinical translation. Moreover, QTPlus‐AM21 exhibited high stability with a similar particle size of 117.9 nm ± 0.75 when stored at 4 °C for 18 h. In addition, 5 freeze‐thaw cycles of QTPlus‐AM21 between −20 °C and room temperature showed little change in average particle size and PDI, indicating high stability of QTPlus‐AM21 (Table S1, Supporting Information).

Much literature showed that treatment with AM21 with LNA modifications could successfully inhibit tumor cell growth in vitro.^[^ ^37^, ^38^, ^39^, ^40^, ^41^ ^]^ However, none of these studies have demonstrated that AM21 therapeutics are effective in inhibiting tumor growth in animal models. Although Nedaeinia et al. first applied in ovo model to evaluate the effect of AM21 in inhibiting the metastasis of colon adenocarcinoma in the chicken chorioallantoic membrane model,^[^ ^42^ ^]^ the antitumor responses by AM21 were hardly measurable, and such in ovo model was not able to represent a real response in terms of tumor growth inhibition by AM21. Later, Javanmard et al. showed that AM21 successfully inhibited melanoma growth in a B16F10 tumor‐bearing mouse model.^[^ ^43^ ^]^ Nonetheless, their delivery platform (MaxSuppressor in vivo RNA‐LANCEr II, BIOO Scientific) containing oil phase and additional small molecules may act as exogenous antigens to randomly stimulate immune cells which can only be used for animal study instead of developing for clinical stages.^[^ ^44^, ^45^ ^]^

In the present study, QTPlus was also able to deliver AM21 to tumor cells and macrophages and exhibit efficient miR‐21 downstream regulations compared with QTsome Original‐AM21 (Figure 2). Although the biodistribution studies in the mouse model showed a major accumulation of QTPlus in liver which is similar to other reports on the biodistributions of lipid nanoparticles,^[^ ^46^, ^47^ ^]^ the significant tumor growth inhibition of QTPlus‐AM21 in vitro and in vivo still make it an effective anticancer therapy. QTPlus‐AM21 exhibited a larger size than QTsome Original‐AM21 (Figure 1A), and the particle size within 200 nm is considered suitable for cellular uptake.^[^ ^32^, ^33^ ^]^ QTPlus also showed a great encapsulation rate for AM21 with N/P ratios from 3 to 10 as demonstrated by gel electrophoresis (Figure 1C) where the capable N/P ratio of QTPlus could be more tolerable than the current LNPs‐based vaccines.^[^ ^15^ ^]^ Taking together, QTPlus with optimized compositions showed significant increases in oligonucleotide delivery in vitro.

### 4.2 AM21 as a Potent Antitumor Agent against NSCLC

In the present study, 16‐mer AM21 was designed to target miR‐21 instead of traditional anti‐miRs with around 20 nucleotides.^[^ ^9^, ^48^ ^]^ This is because mature miR‐21 is often complexed with Ago2 in the cytoplasm where the binding domain between miR‐21 and Ago2 will be the first and 17th to 21st nucleotides from the 5’ end.^[^ ^49^ ^]^ Therefore, a 16‐mer AM21 targeting miR‐21 would bypass the Ago2 binding domains to achieve fully complementary binding with miR‐21 but not anti‐miRs with over 20 nucleotides. The different gene regulation profiles of AM21 in multiple cancer cell lines suggest that NSCLC is the most sensitive to dysregulated miR‐21 biological processes compared with other types of tumors (Table 2). This result might be due to the high expression of miR‐21 in most NSCLC tissues and cell lines based on previous literature.^[^ ^50^ ^]^ Moreover, QTPlus was able to greatly enhance AM21 delivery compared with AM21 transfection in free solution which eventually leads to significant inhibition of A549 colony formation (Figure 3A,B). This is due to the significant Akt1 inhibition and PTEN upregulation as the downstream signaling specifically in miR‐21 inhibition (Figures 2 and 4).^[^ ^51^, ^52^ ^]^ However, it is noticeable that QTPlus‐AM21 was not able to regulate PTEN expression from the mRNA level (Figure 2B,C) but directly from the protein level (Figure 4A,C) when considering Akt1 and Pten as sequential signaling among miR‐21 downstream genes. This might be because miR‐21 inhibition was able to directly regulate PTEN by modulating downstream mediators of PTEN to prevent PTEN from destruction at the protein level.^[^ ^53^ ^]^

Pharmacologically, miR‐21 regulation also shares some signaling pathways which could enhance or remedy the therapeutic mechanisms by chemotherapies such as tyrosine kinase inhibitors (TKIs).^[^ ^54^, ^55^ ^]^ Research showed that treatment with TKIs could recover PTEN expression in lung cancer cells.^[^ ^56^ ^]^ However, EGFR‐mutant lung cancer would develop TKI drug resistance by suppressing PTEN expression.^[^ ^57^ ^]^ Here, treatment with QTPlus‐AM21 could promote PTEN expression and inhibit EGFR levels in A549 cells in vitro and the TME from A549 tumor‐bearing nude mice (Figure 4). In addition, QTPlus‐AM21 could also sensitize A549 cells to erlotinib treatment in vitro (Figure 3C) with a combination index lower than 1 and DRI larger than 1, suggesting that QTPlus‐AM21 and erlotinib showed synergistic anticancer responses in vitro,^[^ ^58^ ^]^ and QTPlus‐AM21 would be a promising anticancer agent alone or in combination with first‐line EGFR‐TKI‐based chemotherapies against NSCLC.^[^ ^59^ ^]^

### 4.3 AM21 Turned Tumor Hot Which Benefits Anti‐PD‐L1 Therapy

Although researchers have demonstrated that miR‐21 deficiency would lead to macrophage polarization into M1 populations and other antitumor immune responses in transgenic miR‐21‐depletion mice,^[^ ^8^, ^21^ ^]^ there is very little evidence showing that ASO therapies could also effectively induce two‐way therapeutic effects targeting tumor and immune system. Here, the QTPlus‐AM21 induced upregulation of CD86 in both human and mouse macrophages (Figure 5C,D) which was further demonstrated to induce macrophage proliferation and M1 polarization by flow cytometry results (Figure 7C,D). Moreover, M1 macrophages stimulated by QTPlus‐AM21 could inhibit MC38 cancer cell growth and wound healing (Figure 7A and Figure S1, Supporting Information) at the same time as QTPlus‐AM21 could directly induce apoptosis in MC38 cancer cell populations (Figure 7B). This effect could be explained by the cytokine‐ or chemokine‐dependent cytotoxicity that the secreted CXCL10, IL‐12, and TNFa in QTPlus‐AM21‐stimulated macrophages could inhibit MC38 cancer cell growth in vitro.^[^ ^60^ ^]^ In the MC38 syngeneic mouse model, 3 mg kg^−1^ of QTPlus‐AM21 showed significant antitumor activity (Figure 8). However, 6 mg kg^−1^ of QTPlus‐AM21 did not exhibit a significantly higher response (Figure 8). This might be due to the upregulated PD‐1/PD‐L1 expression by QTPlus‐AM21 as demonstrated in vitro and in vivo which enhanced the immune escape of tumors(Figures 5A,B and 10B,C).^[^ ^61^ ^]^ Nonetheless, QTPlus‐AM21 treatment to syngeneic tumor‐bearing mice successfully increased CD45+ tumor‐infiltrated immune cells and F4/80+ CD86+ M1 populations in TME (Figure 9), suggesting that QTPlus‐AM21 could turn tumor “hot” with more tumor‐infiltrated immune cells which would be beneficial for additional immunotherapies.^[^ ^62^ ^]^

Indeed, in the same tumor‐bearing mouse model, QTPlus‐AM21/ATZ combination therapy showed increased antitumor responses compared with either QTPlus‐AM21 or ATZ monotherapy (Figure 11). Interestingly, QTPlus‐AM21 and ATZ showed complementary antitumor immunoregulation in TME (Figure 12). QTPlus‐AM21 significantly increased the M1 population which ATZ monotherapy was not able to modulate (Figure 12), and ATZ could significantly suppress Tregs infiltrated into TME which QTPlus‐AM21 was not able to achieve but can only increase CD8 T cell populations along with ATZ (Figure 12). Taking together, the increase in M1/M2 and CD8 T/Tregs ratios, as well as NK cells in the combination treatment of QTPlus‐AM21 and ATZ, would greatly facilitate M1 macrophage activation and antigen presentation to further maintain high T cells or NK cells tumor‐killing functions in TME with minimal interference by Tregs.^[^ ^63^, ^64^, ^65^ ^]^ However, CD11b+ Gr‐1+ MDSCs populations were also increased followed by the combination treatment of QTPlus‐AM21 and ATZ which was solely dependent on the treatment with immune checkpoint inhibitors (ICIs) .^[^ ^66^ ^]^ Considering the significant immunosuppression and tumor progression effects of MDSCs, further MDSC‐targeted ICIs or other immunotherapies deactivating MDSCs should be considered with QTPlus‐AM21. Nonetheless, QTPlus‐AM21 would still be a strong anticancer agent as monotherapy against colorectal cancers or in combination with PD‐L1 ICIs.

## 5 Conclusion

An increasing amount of approved nucleic acid therapeutics has demonstrated the potential to treat diseases by gene regulation in vivo. However, their clinical translation depends on delivery technologies that could improve stability, drug release by endosomal escape, and gene regulation profiles. LNPs provide a lipid compartment for nucleic acid cargos which could sequester from serum nuclease activity and facilitate cellular uptake. In the present study, the QTPlus was utilized to deliver AM21 against cancer. The optimized QTPlus showed significant AM21 delivery efficiency compared with QTsome Original. QTPlus‐AM21 demonstrated compact LNPs structures with high encapsulation efficiency. Pharmacologically, AM21 showed significant miR‐21 downstream gene regulation and was able to polarize macrophages into the M1 population in favor of antitumor immune responses. In the A549 cell line, QTPlus‐AM21 not only showed significant antitumor activity but also sensitized tumor cells to erlotinib cytotoxicity. Moreover, QTPlus‐AM21 could also turn tumor “hot” by modulating macrophage populations in TME. Lastly, QTPlus‐AM21 showed significant antitumor responses in vivo in combination with ATZ. QTPlus‐AM21 greatly increased antitumor immune cells infiltrated into TME which shed light on its great potential as an adjunct therapy with first‐line ICIs.

## Conflict of Interest

J.L. and F.S. are employed by Zhejiang Haichang Biotechnology Co., Ltd. Y.H. and X.Z. are employed by The Whiteoak Group, Inc. The authors declare that the research was conducted in the absence of any commercial or financial relationships that could be construed as a potential conflict of interest.

## Author Contributions

R.J.L., Y.H., and Z.Z.: conceptualization; Z.Z.: methodology; Z.Z. and J. C.‐T.K.: software; Z.Z., J.L., F.S.: validation; Z.Z. and Y.H.: formal analysis; Z.Z.: writing—original draft preparation; Z.Z., Y.H., Y.H., X.Z., and R.J.L.: writing—review and editing; R.J.L. and Y.H.: supervision; R.J.L.: funding acquisition. All authors have read and agreed to the published version of the manuscript.

## Supporting information

Supporting Information

## Data Availability

The data that support the findings of this study are available from the corresponding author upon reasonable request.
