# Supplementary material for: Antitumor Activity of Anti‐miR‐21 Delivered through Lipid Nanoparticles
Source: Adv Healthc Mater. 2022 Dec 9;12(6):2202412. doi: 10.1002/adhm.202202412 (PMC11468686; doi:10.1002/adhm.202202412)
Supplement: Supplementary file 1 — Supporting Information [file ADHM-12-2202412-s001.pdf]

ADVANCED HEALTHCARE MATERIALS

Supporting Information

for *Adv. Healthcare Mater.*, DOI 10.1002/adhm.202202412

Antitumor Activity of Anti-miR-21 Delivered through Lipid Nanoparticles

*Zhongkun Zhang, Yirui Huang, Jing Li, Fei Su, Jimmy Chun-Tien Kuo, Yingwen Hu, Xiaobin Zhao and Robert J. Lee**

Table S1. Stability of QTPlus-AM21 for multiple freeze-thaw cycles from -20°C to room temperature.

Freeze-Thaw Cycle	Mean Particle Diameter (nm)	Particle Diameter STD	Average PDI
0	117.5	0.75	0.019
1	119.7	1.29	0.022
2	120.4	1.04	0.037
3	119.5	0.78	0.035
4	117.8	0.40	0.039
5	118.9	0.53	0.102

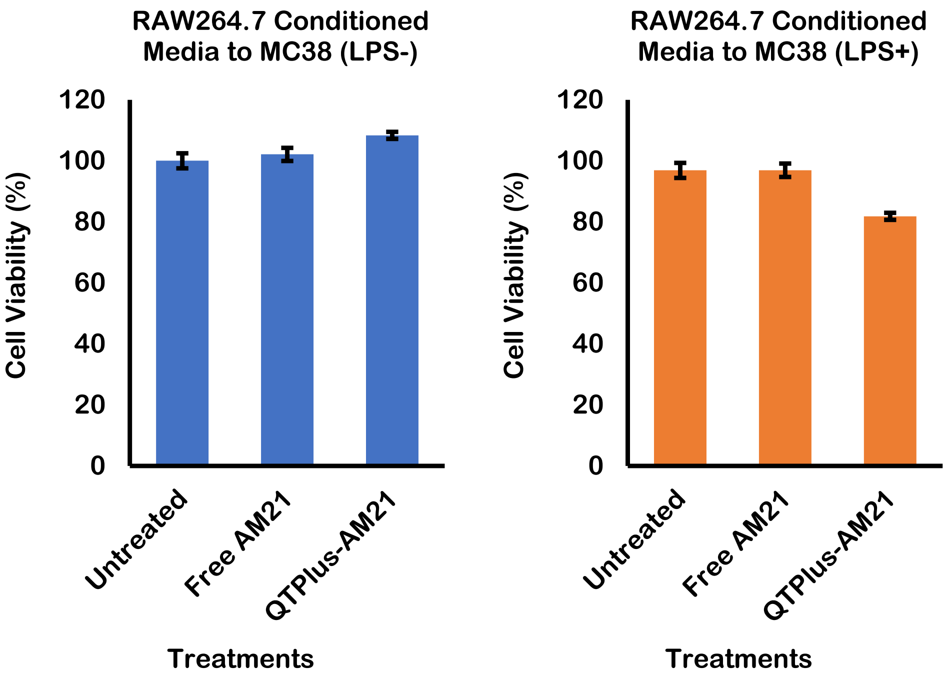

Figure S1. Cell viability of MC38 treated with conditioned culture media from RAW264.7. Briefly, 3x 10^5 of RAW264.7 cells were treated with free AM21 or QTPlus-AM21 for 24 hours in the absence or presence of 1ug/ml of LPS. The conditioned culture media from RAW264.7 cells were collected and used to treat MC38 cells for 72 hours. The untreated control in MC38 cells were incubated with conditioned media from untreated RAW264.7 cells. The cell viability of MC38 treated with conditioned media from RAW264.7 was performed by MTS assays using CellTiter 96® AQueous One Solution (Promega, Madison, WI) per manufacturer's protocol.

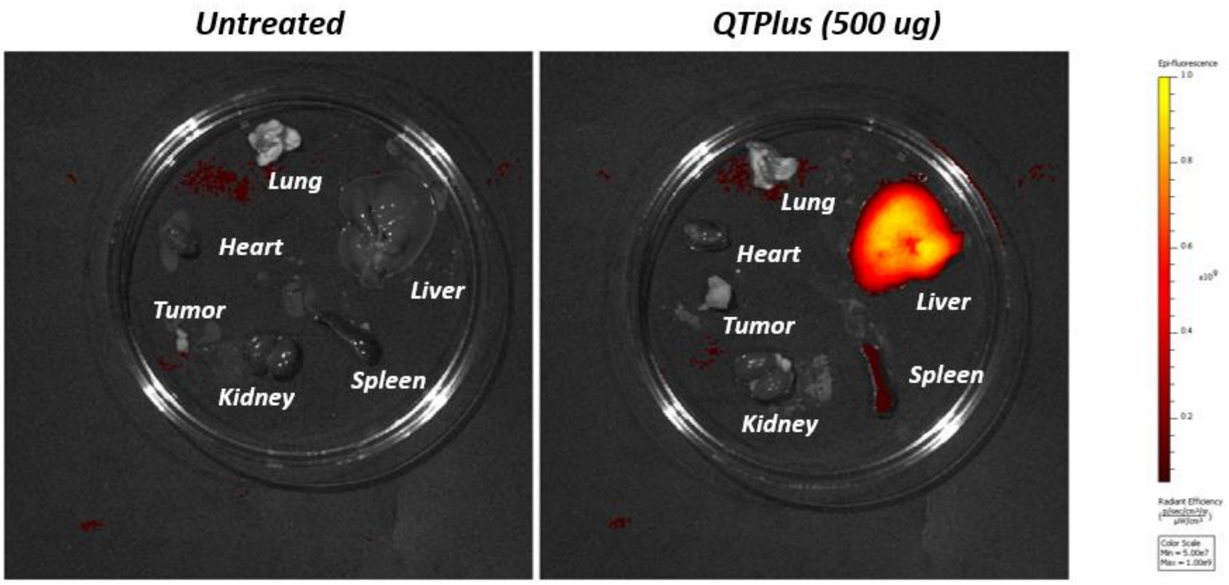

Figure S2. Biodistribution of QTPlus. Briefly, 500 ug of QTPlus encapsulating 16-mer ASO labeled with 1% DiR lipophilic tracer was intravenously injected into nude mice 24 hours prior to the necropsy and imaging. Organs from untreated and treated mice were collected and imaged under IVIS Lumina II (PerkinElmer Inc., Waltham, MA, USA) with an excitation wavelength of 710 nm and an emission wavelength of 814 nm.

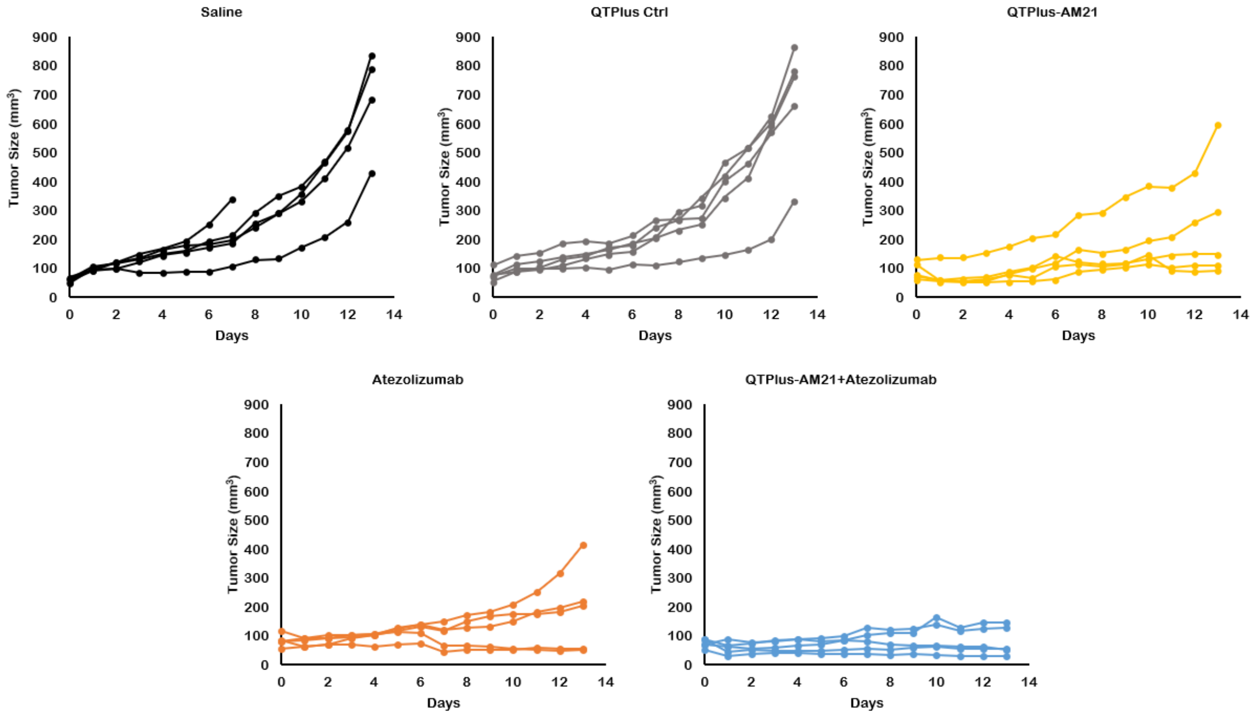

Figure S3. Tumor growth on individual C57BL/6 mice treated saline, 3mg/kg QTPlus-Ctrl, 3mg/kg QTPlus-AM21, 10mg/kg ATZ, and QTPlus-AM21/ATZ combination (3mg/kg QTPlus-AM21 and 10mg/kg ATZ) every 3 days for 5 doses.
